# False positive bladder scan in ascites with anuria

**DOI:** 10.1002/ccr3.2281

**Published:** 2019-07-03

**Authors:** Sarosh Janardanan, Ahmed E. M. Moussa, Philip James

**Affiliations:** ^1^ Ashford & St Peter’s NHS Trust Hospital Chertsey Surrey UK

**Keywords:** ascites, bladder scan, suprapubic catheter, urinary retention

## Abstract

Urinary retention is commonly diagnosed based on history and examination along with bedside bladder scan. However, in patients where clinical examination is unreliable (patients with obesity, anasarca, and ascites) and diagnosis is uncertain, the bladder scan findings should be interpreted with caution and definitive imaging is mandatory before further intervention is instituted.

## 1 CASE REPORT

A 87‐year‐old man was admitted under the medical team with cellulitis. His comorbidities included hypertension, moderate tricuspid regurgitation, chronic anemia, bilateral fracture neck of femur due to recurrent falls, coronary artery bypass surgery, and cirrhosis. He mobilized with assistance and had a BMI (body mass index) of 42.

On examination, he had shortness of breath with bibasal crepitations. His abdomen was soft, nontender but distended. He had generalized edema. His blood investigations showed normal renal function.

He was on antibiotics for his cellulitis and was being treated for possible fluid retention secondary to heart failure. To accurately monitor his urine output, a catheter was inserted but no urine was drained despite bladder scan showing a large residual in excess of 1 L. A gentle lavage was done which did not encounter any resistance or change in the color of the effluent. At this point, a urology referral was sent for a suspected false passage in the background of urinary retention.

**Figure 1:**
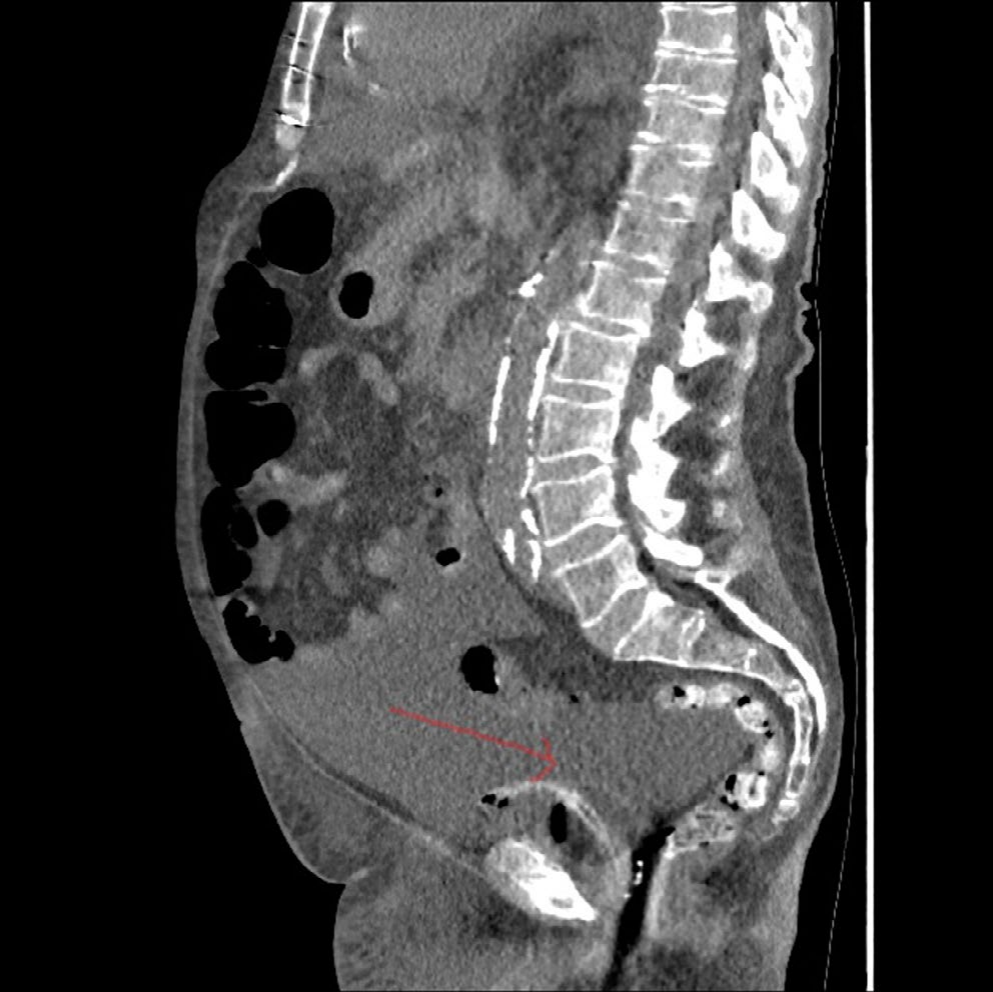
Catheter noted in a decompressed bladder (red arrow)

**Figure 2:**
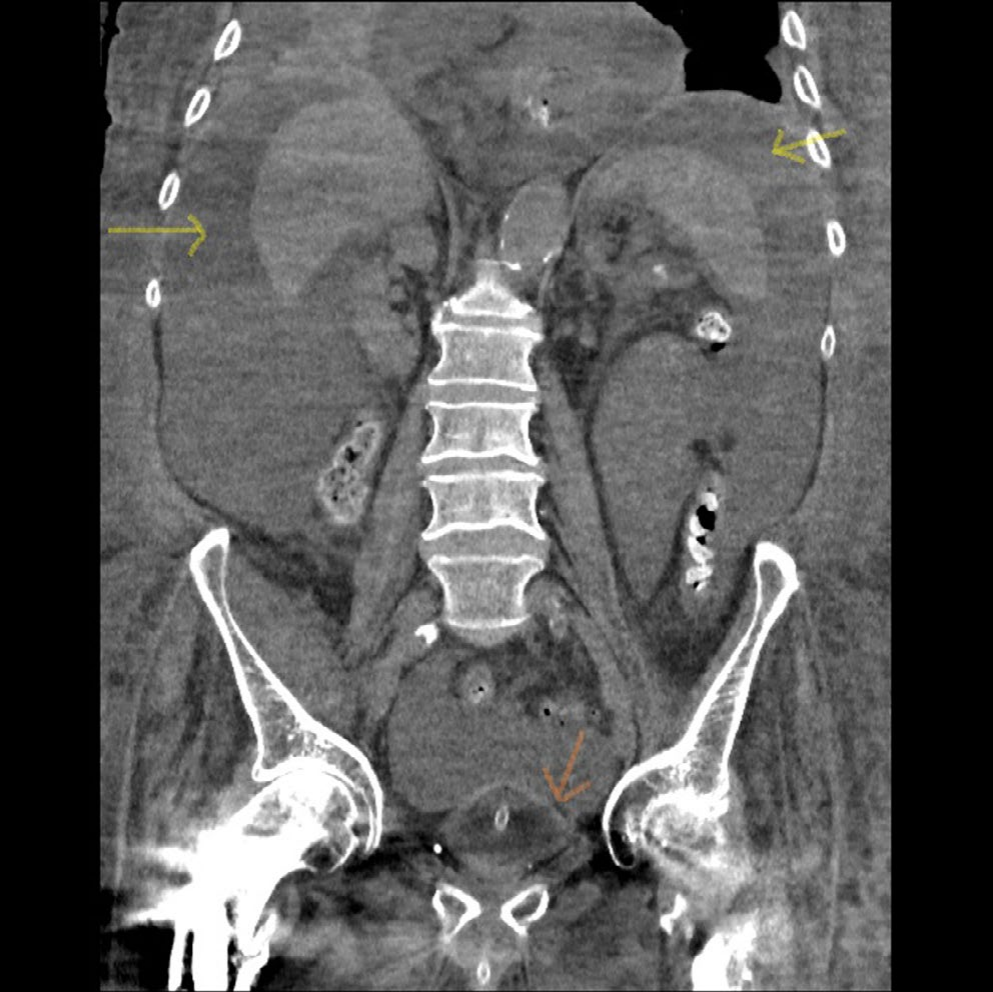
Gross ascites (yellow arrow) with empty catheterised bladder (orange arrow)

The urology team reviewed the patient and changed the catheter. The catheter insertion was found to be uneventful; however, no urine was drained on insertion. Clinical examination was difficult due to body habitus and anasarca. It was decided to get imaging to confirm findings. The CT findings (since out of hours sonologist was not available) showed a decompressed bladder (Figure 1) with a properly sited Foley catheter along with the presence of moderate ascites (Figure 2). The patient was managed for his heart failure and anuria medically.

## 2 DISCUSSION

Urinary retention is a commonly encountered problem based on clinical findings and a bladder scan.^1^ However, in obese patients or in presence of subcutaneous edema, the bladder may not always be palpable. Similarly, patients with chronic urinary retention and neurogenic bladder are often unaware of the problem due to the absence of pain or discomfort.^2^ The bladder scan is sensitive for picking up any fluid in the abdomen but not specific for urinary retention. This should be kept in mind while interpreting the results especially in patients who are at high risk for having ascites. Other causes reported in literature for a false positive bladder scan are ovarian cyst, renal cyst, and uterine myoma with cystic degeneration. The overall false positive rate of bladder scan for urinary retention has been reported as 9%.^3^ Insertion of a suprapubic catheter in these patients without prior imaging can lead to serious complications like bowel perforation, vascular injury, or peritonitis. Since the appearance of ascitic fluid is very similar to urine, this may give false assurance of diagnosis in the unsuspecting clinician. The logical approach to deal with the above problem is to flush the catheter with sterile water. If no resistance is encountered and effluent is clear, get formal imaging to confirm site and consider alternate diagnosis. On the other hand, if resistance is encountered and the effluent is blood stained, remove the catheter and get urology opinion.

## CONFLICT OF INTEREST

None declared.

## AUTHOR CONTRIBUTIONS

SJ: involved in writing up the article (main presenting author). AEMM: involved in gaining patient consent and conducting a literature search (second author). PJ: put forward the idea of writing this article up along with providing feedback at every step (third author and the catheter project lead of the department).
